# The Needs of the Medical Profession

**Published:** 1872-03-15

**Authors:** Norman Bridge

**Affiliations:** President of the Association


### THE NEEDS OF THE MEDICAL PROFESSION.

AN ADDRESS DELIVERED BEFORE THE ALUMNI ASSOCIATION OF THE CHICAGO MEDICAL COLLEGE, MARCH 12, 1872.

BY NORMAN BRIDGE, M. D., PRESIDENT OF THE ASSOCIATION.

The annual address before an Alumni Association seems almost a useless formality. It is our habit to expect certain things to be said, and said in a certain way. We look for congratulations that another year finds us together, and that another class is added to the alumni; for the statement that the influence of our Association ought to be greater; and that the members must be faithful to the organization; for a few reflections on our general working; and above all, we look for the information that our *alma mater* continues faithful.

We expect, too, that the address will, after apology for its own weakness and thanks for the honor conferred by allowing it to be made, send the few of us who gather at the annual reunion, back to our homes with benediction.

Let me say just as few such words as possible.

The aims and objects of this Association I need not stop to tell—every member understands them. Nor would words of mine induce members to act differently toward the organization than they have in the past.

We have now been in existence five years, and ought to be no stripling. Our membership numbers hundreds, and we have done some things which even *we* may call commendable. One, surely, has been offering a prize for an essay—the apathy on the subject of writing having proved our need of such a measure; another, perhaps, is the compiling of a necrology of our alumni, however much or little we have done for our *alma mater*. Three times has a prize been offered for the best essay—the first year it was not awarded for want of competing essays written according to the rules; the second year only two essays were presented, and this year not one.

How far this matter has come to look trivial by such neglect; what it indicates for the scientific and literary taste of the alumni, the Association may judge.

Shall we continue this offer of a prize, and hope that next year and the years to come more members will write?

I think we should.

Some of the most valuable of all contributions to scientific knowledge have been given to the world as prize essays. There is no considerable body of medical alumni anywhere who would not spurn the insinuation that no men in their ranks have ability to improve our literature and knowledge by their labors. The best minds in the profession have not refused to enter the lists for such a badge of excellence as this, but have been glad to compete for it, less, be it said, for its intrinsic value, than for what it stands for. Let us then offer the prize on the same conditions as heretofore.

If our help to the school of medicine that graduated us has been little, we may question the propriety of retaining this as a stated object of the Association; for the school is taking long strides in the right direction, whether our hands be pushing or not.

The child is always prejudiced in favor of his mother; he magnifies her virtues, and refuses to see her imperfections. We are exactly like other children. But I am sure that year after year substantial progress can be noted in the more peculiar work our benign mother started out to do.

Established as she was for an idea, to make practical and make a force of that principle her founders before and since have faithfully battled for, by slow degrees, one after another, the medical schools of the nation are incorporating that idea into their curriculi and requirements.

I do not say all the increased thoroughness in Medical College instruction of the last half-dozen years, is due to the example and influence of our *alma mater*, but nobody can deny her a major share of the credit of such progress. To say she never errs is to make her infallible. But if she does now and then a silly or an unjust thing, what of it! She is the foremost exponent this country has of advanced ideas in medical education, and in the work actually done for the elevation of the standard of all good qualities in medical men, she stands to-day head and shoulders above all other institutions on the continent.

But if reforms have been made, others are needed. The medical profession is not perfection; it does not come up to the beau-ideal of any man ; as a whole it may equal the example many doctors give us, but any one who takes the trouble to think what the profession ought to be, is sure to put the standard higher than its present average.

Then there is chance for improvement, and let any words of mine to those present here to-day, be a candid talk on some of the evils in the profession and some of their possible cures.

It is never a difficult task to criticize; fault-finding especially has always been an easy, albeit usually a mean business. It is, moreover, easier to tell a hundred bad things of a class of men, than suggest a sure way of curing one. But the gentlemen to whom these words are addressed are the very last in whose ears I would glorify the profession, and it has seemed to me profitable at this time to review some of the defections of our brotherhood, some of the bad things to be got rid of in order to become better.

Of course we all agree that the most pressing want of the average American doctor today is more knowledge about his business—more drill and thorough schooling in medicine. He needs to attend more lectures, read more books, and above all to have more opportunities for clinical study and observation ; he needs to see more actual practice where he can study it, before he begins practice for himself and alone.

But the want of more thorough medical education, the need of more rigid requirements by the Colleges, a longer course and more terms, have been of late so thoroughly discussed that they are well understood, and I may pass them by.

Another of our great needs is more learning outside the medical sciences. One of the learned professions—if we care to accept or retain that title the world has seemed ready to accord us—one especially into the daily work of which such diversified problems enter, which deals with such vital interests as the medical profession does, should be made up of men of greater knowledge and broader culture than the average of American physicians. A physician, I think no one will deny, should have a degree of knowledge in all departments of general learning, especially all sciences, a general culture and taste second to no other professional man ; he ought to have a sum-total of knowledge greater than any University is able to teach him.

Liberal education, whether gained in or out of school, separates the profession into two classes, of scientific men and empyrics. It not only, by the correct appreciation it gives a man of the weal of the profession, helps him to be an honorable member of it and not a charlatan from intention, but it frees him from liability to believe in humbugs and so be a quack from ignorance.

And in a profession like this, into the study and practice of which so much delusion and imposition always find their way, and with such ease, there seems to be nothing that can free a man from belief in such things—unless he has a kind of common sense so rare that few men possess it—except the influence of study.

Now this sounds, I know, like a school oration on the value of learning. But we cannot dodge the fact that medical men in this country are, many of them—the majority of them—deplorably ignorant, given to beliefs in direct conflict with both science and good sense, as well as practicing upon the people even more humbugs than they themselves believe in. So true is this statement, that in many parts the profession is held in practical disgrace. The term "Doctor" has come to mean either something supernatural and the gift of strange and unusual insight into human bodies, or to suggest a quarrel about matters it ought to be easy to understand among a set of men bigoted, conceited, and naturally jealous and combative.

A per cent. I would not like to name are horse-jockies and intemperate men ; half of them know nothing of the primary branches and little more of the practical branches of a medical education, above what they gained in their scant three years of time partly devoted to study before graduating ; and certainly half the physicians in this country could not hold an intelligent conversation with a person of high culture, on topics which such a person would most delight in.

I once knew a man, a graduate of a New England Medical College, who had a fair standing as a physician in his neighborhood, who tried to make me believe that the weather of the last two days of each month was the index of the weather of the month following, and offered as proof to let me watch the point for a year and see if it did not come true. A decent degree of natural knowledge would have made it impossible for this man to believe such absurdity ; yet he was a type of a class by no means small.

The profession, in ages past, has had so many senseless beliefs in matters of physiology and therapeutics, that we laugh at them. Yet we believe in this age that the sight of a bleeding throat by a pregnant woman will stamp a mark across the neck of her child, to say nothing of the belief in the efficacy of a thousand-decilionth grain of something as a medicine.

Out of this odium, from beneath this cloud, broad culture alone can lift the profession. But how always to gain this is less easy to say.

All agree it may best be begun with a four years' University course ; doubtless it would be better if every physician was a graduate in such a course before his medical studies begin.

Although this may furnish often very questionable practical material for help in everyday life, it is acknowledged the best way in which to get well grounded habits of systematic thinking. And the course of those Medical Colleges, among which is our *alma mater*, that have made it to the advantage of all their students to have attended a University course, cannot be too highly commended.

But few medical students are able to begin their education with College drill, and many of us must do without any. Many of us come to our medical study direct from the farm, the workshop or store, unused to study, and here our first long schooling is received. We graduate and go out and locate, possibly with hands full of practice, certainly with minds full of the science of getting a livelihood ; and from a very inconsiderable amount of work at first, which seems great, our cares grow from year to year, and we feel unable to devote more time to reading than will partially keep us posted in the literature of the profession and in the daily newspaper gossip. So we go through life with little addition to the stock of general knowledge we started with, and grow every day more fossilized. There seems, then, no way for nine-tenths of us to gain the knowledge and culture it is conceded we ought to have.

Now this would be true if the premises were not false. Possible we could not study general subjects before graduating in medicine; but it is false, that if we have a taste for such things, we can find no time to pursue them afterward.

The fault lies in the bad economy of time and in the want of inclination, chiefly the latter. Those who have strongest taste for books and study somehow do find time for both, although they do more work than we who complain of our burdens ; they learn to use the moments that we waste.

Did only the right sort of men—namely, students—study medicine, voluntary self-culture would be for the profession all-sufficient. But men take up medicine on all pretenses ; some blindly thinking it will make them rich; others for its supposed honors, and because it is expected to furnish a long rest from much work. These are more foolish than the first, for they fogret that honors come only with work, and that a long rest means the sin of laziness. I knew a man who studied medicine chiefly because he liked to drive horses, and he knew that in a country ride his longing could be satisfied. He studied eight months and began practice.

Now we submit that no man should enter the profession who has not an innate love of science, of books—an ambition to know more and more, and to know the reason why of everything.

*Here is our great professional crime!* Physicians who, without this consideration in view, advise men to study medicine, who advise all to do it who would like to become doctors, are most surely culpable, and on their heads must fall the weight of obloquy cast on the calling. And my words to the graduating class of to-day, if they are worth anything, would be these: That if there is a man among them who is not so made up that he will, that he must, spend his spare time in study, to be each successive year a better balanced and a wiser man, my advice to him is to take his sheep-skin home as a curiosity, keep it securely, but not to hang out his sign or write a prescription while he lives.

Physicians should be life students, students of everything; they are not to-day, save in small minority. This fact gives ground for the sneers against the profession, for half the quackery, and is the reason more sick folks do not recover. In regular practice—the only broadly eclectic practice—it operates with special injury, making the work of the ignorant who handle double-edged tools and cut at random, show in bad contrast with that of such quacks as are shrewd enough to pursue an expectant course.

The only sensible remedy that I can think of, for this, our greatest drawback, is to stop making doctors who will not be students. Those whose views coincide with this may do something to elevate in this direction the profession now in existence; but men who dislike the closet cannot be made to like it, and our greatest good lies in guarding the entrance to the ranks of the profession, not in attempts to change men after they get in.

Another vice to be remedied is the flagrant want of generous conduct of practitioners toward each other; the lack of pride in the profession, its dignities and amenities. We want more *esprit-de-corps*, more ethics, more code of ethics. It has been said no association can make ethics for any man. That is true, but a body of men may agree as to certain principles of correct conduct that *are* ethics, which every member may live by conscientiously. It is contrary to the idea of a profession not to have such a rule; only by fidelity to establish good usage can opinions of reliability be engendered in others toward us. Could the American code—the acknowledged paragon of all written statements of medical ethics—be faithfully adhered to by all physicians, the medical, despite its lack of erudition, would be held for honor and magnanimity second to no other profession. Ethical conduct, what is it? Only more than the demeanor of a gentleman; the abandonment of all that is ungenerous and unjust.

We must be taught somehow that the profession of medicine has a duty society inexorably demands of it. That duty is in correct conscience to conserve human happiness—by improving human health. While the practitioner must have a living and full praise for whatever he does that is worthy, he has no right to gain or accept either to the neglect of this clear duty; if he cannot stay in the faculty on the basis of duty, he has the alternative to seek other avocation.

To teach this principle, ethics must be understood, and every Medical College course ought to be the means of teaching to students this American code. Men have been actually graduated in this city without being aware such a code exists; whereas every one should leave college with this document—so clear and simple, so just and sufficient—if not committed to memory, surely with its principles as fixed in mind as his own name.

But men do not worship the code greatly, nor obey it; most do the opposite.

Average it in city and town, and jealousies and bad faith have done as much to injure the profession as all its ignorance. It has come to be a wonder in certain parts for doctors to agree on anything, or to speak well of each other. When they do commend each other, it is surmised they have some interested motive, some ulterior purpose to serve; when they slander each other's professional character and knowledge, they are supposed to be in their normal condition. Their work has grown into a petty warfare to find which man can convince most patients that he and not his rival is to be trusted.

Doctors ought in a sense to be their own judges. Every pure man covets the good opinions of others, but we value the opinions of those chiefly whom we know capable of judging us rightly. No true artist cares for the strictures of a boor on his work; neither would an essayist listen to the criticism of an ignoramus; those only of similar capacities can command our full respect by their opinions. And no man working in any field of worthy endeavor in the past, in literature, art or science, has been satisfied with himself unless he has gained some plaudits of his only competent judges.

How can the faculty of medicine be an honor to the age so long as half its members disregard or laugh at the opinions of the body as a whole, or the best part of it? Scores of them do this, and many an one acts apparently with the idea uppermost that every other doctor is his natural enemy, and he is no friend to any of them; he cares neither for their condemnation or praise, unless perchance the latter may sometimes bring him a fee. Not one such man has ever done a thing to make the profession a body to which one of fine sensibilities would like to belong; their influence has always been to lead minds in the direction of charlatanry, and to prevent any united effort and progress; their example has invited the profession to become a *trade*, the chief work of which shall be to make money and influence, in which every man shall try, regardless of means, to outstrip his neighbor, leaving to the devil the hindermost.

All the advances the profession has made have come through those who have aimed to make it such that students and good men might like to enter it. There have been men—and heaven help us add to their number—who, believing the profession should be nothing if it is not everything honorable, have lived by a high standard of ethics toward both practitioners and people; who, while studying to take a high place, have helped others up instead of pulling them down; who, in seeking to accomplish much, have had some charity for the shortcomings of others; who have carefully avoided word or deed that would make the brotherhood less worthy, less united and fraternal. They have somehow learned the truth that he who with malice aims a blow at another strikes hardest himself, and so have not struck at all. Into their professional life they have thrown somewhat of the sentiment of treating others as they would be treated—which, after all, is the groundword of that code of conduct we boast of. To such men we owe praise that the faculty is no worse, and with such men of the future lies the task of making it still less deserving of censure.

But to accomplish these good aims there is one thing practitioners seriously need; we all need it as a great help, less for what it is than for what it will aid us to do and to be; that is more close social and professional relations with each other.

Medical societies are a good thing both for knowledge and good feeling; with frequent meetings they keep us awake to the science and literature, and beget a spirit of emulation always wholesome. It is the feeling of every progressive physician that he ought to belong to a medical society.

Practitioners are too much isolated, they feel too little the force of each other's influence and existence. Isolation breeds exclusiveness, jealousy and selfishness; open communion, free mingling with others, alone makes us broad, charitable, cosmopolitan. Bring medical men face to face often, and all there is in them that makes human nature narrow and base would slink away for shame. There have been enthusiasts who think medical societies are a panacea for all professional ills; that if we would only all join them, then, to borrow the language, "we might all be happy."

At least this is true, that the present system of societies, completely organized, holding stated meetings for transaction of business and for discussion of scientific questions, is the only and so the best means generally undertaken in this country for bringing practitioners together to measure ideas, and for social intercourse. This system is good, and because it is organized, should never be abandoned. Yet many are dissatisfied with its formalism—its stiff parliamentary machinery. They complain that too much time is consumed in discussion of points of order, and too little to scientific work. I know dozens of physicians who, for this reason alone, do not attend once a year medical societies to which they belong.

Moreover, such societies are never formed except in the larger cities, placing them practically beyond reach of the majority of practitioners.

It has been a study with some, in time past, to improve on this system. A few months ago two men in this city put their wits together to solve this problem, and such a solution as they have reached I will describe.

Their aim was to supplement the present societies by something that would supply a want which *they* could not, while their acknowledged work should not be disturbed; to utilize powers and elements of mind the societies could, in their nature, never command.

They have, as the result of their thought, brought forth a sort of medical society which, by what it has done so far and seems capable of, I think commends itself to the consideration of the profession.

I am so fully persuaded of its value, so sure of its benefit to those who have been favored with its privileges, that I must ask to describe it.

I would not dare put it forward as a new measure of reform, had its qualities not been tested by time; but it is now near two years old, it has had no drawbacks, but from the beginning a lusty growth—somewhat in size but more in strength—and gives promise of a long life.

I call it a *sort* of a medical society; but whether it is properly styled a society, a club, a sociable, or a meeting, I am at loss to decide.

It has no name, no constitution, by-laws or records; no officers or order of business; it has no regular or stated membership; it exacts no scientific duties of its members; it is never called to order, nor does it proceed in a parliamentary way; it is absolutely and in every particular informal. It does not know, save from meeting to meeting, where it is to be harbored. Its whole life rests in the understanding, clear and positive, of its members of just three things: That the meetings shall occur in the evening every two weeks, at the homes of the members, alternating; that conversation shall be the order, but that no formality shall be tolerated; and that there shall be in the course of the evening some very simple refreshments.

These principles were never put in writing; not all at once were positively agreed upon; but as they have seemed to work well, they have grown into the acceptance of the members until their infringement has come to be next to impossible. So every fortnight these men—a dozen and a-half—sit down together and spend an evening in conversation on any subjects that may please them best. There is no restriction; there is no saying Mr. President; every one is at his ease and is always in order. No extravagance or display is countenanced in refreshments, and alcoholic liquors are rigidly excluded. At whatever hour, then, the members find their homes, they are always refreshed and always sober.

Now, conversation is the most valuable means of communion; men tell here their best ideas, and say them just as they are, and here they think their best thoughts and break them to others; here they are, more than anywhere else, without hypocrisy—they act themselves. In these meetings of conversation, men get the cream of each other's minds, which comes with a frank expression; here men are found to be overflowing with knowledge and good thoughts who, could they be heard only in a formal meeting, would never speak, and they let go their gems of wisdom so silently and unconsciously they are not even made diffident by themselves knowing it. Men are heard here aright who, in an unwritten speech, could not fail to be misconstrued. Then these men are obliged to shake hands with each other, and to look each other in the face, and they know this must be repeated every two weeks; and if there were no other reason, this would compel them through the fortnight to be courteous to each other and all the profession. Men dislike to ask each other's pardon, and one will use twice the effort to avoid the necessity that he will in arousing moral courage to do it. Add to this the fact that the whole society must sit down and entertain each other at a little feast, and you have all the elements needed to cultivate the best professional feeling and understanding.

And say what we will, we cannot deny the value of having something to eat at a social meeting of any kind. However simple, it makes the meeting less formal, and brings all the party nearer together; it makes them more kindly and considerate.

There is no way to make a man good as sure as to make him humble, and there is no way to make him humble so unfailing as to make him feel his need. As he bends to receive from mother nature her gift of food that nourishes him, he is a debtor and he feels it, whether he knows it or not. In the act and mood of receiving he banishes much or little of the acerbity and venom he has, and his heart grows warm as his body feels refreshed.

The benefits from this measure are confessedly great. It has made, with the other features of the meetings, this nameless, unwritten society a marked success. It is the unanimous verdict of the members that the meetings are the most profitable they have ever attended, both in a professional and in a social way.

Is this idea of a society worth copying? I think it is. Its value in a city is undoubted; in a sparsely settled neighborhood it might be far greater. Societies not unlike this could be maintained in any place where there are two doctors—provided all the physicians would unite on it—which cannot be said of the old societies. By such all the good gained by the project in Chicago could be realized, while they would have the advantage over us that, farther removed from association of numbers, living where the fields of labor of practitioners invariably run into each other, and men are exceeding liable to hit each other's elbows, the society would be enhanced in value in proportion to the lack of the professional advantages large cities can furnish. This sort of a club could exist where, on account of numbers, one of the organized societies could not be thought of; they prosper only with large numbers, this prospers best with few.

Could the two doctors, the only representatives of the profession in any town, fail to be friends and champions of progress if they would resolve to take dinner and a long talk with each other every two weeks, and carry it out? No, but at such a spectacle the people would open their eyes with wonder; they would repeat again the words of the lion and the lamb, and would hail this as the harbinger of a new millenium.

